# Focus on biomarkers, confounders and new therapeutic approaches in trauma

**DOI:** 10.1007/s00068-022-01976-1

**Published:** 2022-06-13

**Authors:** Dirk Henrich

**Affiliations:** Department of Trauma, Hand, and Reconstructive Surgery, University Hospital Frankfurt, Johann Wolfgang Goethe University, Frankfurt/Main, Germany

Dear Reader,

It is a great pleasure for me to present the current special issue of “Focus on: biomarkers, confounders and new therapeutical approaches in Trauma”. This special issue summarises basic research and reviews on the topics of scoring systems and biomarkers, the confounder alcohol and novel therapeutic approaches.

A rapid and reliable assessment of trauma severity and involved organ systems is essential for the assessment of complications, outcome and treatment strategies. Therefore, research into biomarkers and scoring systems is of great importance for the trauma surgeon. At the same time, it is important that factors that temporarily influence the physiological status of a person, such as alcohol consumption, are also included in therapy decisions, as they can lead to surprisingly extensive changes in vital physiologic processes and thus have a significant influence on the outcome of the patient. Ultimately, new therapeutic concepts must be tested experimentally, as this is the only way to continue to make progress in the treatment of severely injured patients. The articles compiled here show in particular the broad range of current research and also provide new impulses for the assessment of (organ specific) trauma and the development of new treatment concepts.

Biomarkers as well as score systems are an important tool to assess the clinical course and outcome after major trauma. While trauma scores consider the whole organism, organ-specific biomarkers can help to assess the damage or recovery of a specific organ much more precisely. However, currently available biomarkers often times lack the desired accuracy, so it is necessary to search for alternatives. Current research focuses on extracellular vesicles, µRNAs and tissue-specific proteins. Extracellular vesicles (EVs) are naturally released from almost all types of cell. They contain cell- and tissue-specific µRNAs and proteins and carry specific surface receptors that indicate their cellular origin. This makes EVs of great interest as biomarkers. In a review article, Weber et al. summarise the current state of knowledge on the regulation of EVs and their diagnostic value under trauma conditions [1]. The relevance of the neutrophil-derived long noncoding (lnc) RNA IL-7R for the development of MODS after trauma is investigated by Jin et al. They report that lnc RNA IL-7R is an independent predictor for the development of MODS and that this RNA is significantly decreased in patients who develop MODS [2]. The significance of the protein-based biomarker neurofilament light chain (Nfl) in patients with concussion or head injury was investigated in a systematic review by Karantali et al. The analysis showed that serum Nfl levels were significantly elevated in patients with concussion compared to healthy subjects, and that sports-related concussion in particular was associated with higher Nfl levels. Furthermore, it is concluded from the studies that further studies on Nfl in mild TBI are needed [3].

The trauma-induced pathophysiological processes are not only dependent on the injury severity and pattern, but are also significantly influenced by substances such as alcohol. Two papers at the same time have this as their subject, under different aspects. Stettler et al. investigated the viscoelastic properties of the blood of injured patients as a function of the blood alcohol level. It was possible to associate a high blood alcohol level with an increased incidence of fibrinolysis abortions, which should be taken into account in haemostatic resuscitation after injury, as these patients could be harmed by antifibrinolytics [4]. The alcohol-induced physiological changes, probably due to increased barrier disruption of the intestinal epithelium, lead to a significant increase in systemic endotoxin activity, as Sturm et al. showed in a prospective study. Interestingly, the effects were significantly less pronounced in female subjects [5].

The release of endotoxins into the circulation through trauma-induced mucosal barrier disturbances leads to further activation of the immune system. Therefore, it makes sense to develop forms of therapy to protect the intestinal tissue from reduced blood flow and ischaemia. Aletti et al. were able to demonstrate in animal experiments that continuous enteral protease inhibition by tranexamic acid (TXA) led to a significantly improved outcome after haemorrhagic shock [6]. Such preclinical results need to be confirmed in prospective clinical trials to justify clinical use. Whether early TXA administration in polytrauma patients with TBI involvement has positive effects on outcome was investigated by van Wessem et al. However, in a large-scale prospective cohort study with more than 200 severely injured patients included, no significant positive effect of TXA application could be demonstrated [7].

After initial life-sustaining procedures, the restoration of injured tissue after trauma is the goal of trauma surgery. However, the surgical procedures that are often necessary for this purpose represent a further stimulus for cellular immune components, as Teuben et al. and the TREAT Research Collaboration showed in the polytrauma model of the pig. Unilateral intramedullary femoral nailing led to a significant activation of circulating polymorphonuclear granulocytes (PMN), which were not increased in concentration but in their CD11b expression over the 72-h observation period compared to the control group (polytrauma without femoral intramedullary nailing) [8]. This finding again shows why it is particularly important to improve existing therapy options for the surgical treatment of trauma sequelae, or to develop gentle new therapy options. The use of proangiogenic cells to improve wound healing is a promising procedure from this point of view. Sommer et al. investigated whether the systemic application of endothelial progenitor cells (EPC) is equivalent to the local application of these cells into the wound area. Using the ear wound model of the immunocompetent, hairless mouse, it was found that EPCs led to significantly accelerated wound closure with associated increased vascularisation activity. Moreover, equivalent healing results could be achieved with local application using only 10% of the systemically applied EPC number [9].

I hope you enjoy reading the articles on "Focus on biomarkers, confounders and new therapeutical approaches in Trauma” and that they will inspire you to develop and evaluate new ideas and treatment concepts.

With kind regards.

Dirk Henrich.
